# A New and Rapid HPLC Method to Determine the Degree of Deacetylation of Glutaraldehyde-Cross-Linked Chitosan

**DOI:** 10.3390/molecules28217294

**Published:** 2023-10-27

**Authors:** Ons Amamou, Jean-Philippe Denis, Élise Heinen, Taoufik Boubaker, Sébastien Cardinal

**Affiliations:** 1 Département de Biologie, Chimie et Géographie, Université du Québec à Rimouski, Rimouski, QC G5L 3A1, Canada; 2 Laboratoire de Chimie Hétérocyclique, Produits Naturels et Réactivité (LR11S39), Faculté des Sciences, Université de Monastir, Monastir 5000, Tunisia

**Keywords:** glutaraldehyde cross-linked chitosan, degree of deacetylation (DDA), potentiometric titration, derivatization with DNPH, HPLC

## Abstract

Chitosan is a linear biopolymer composed of D-glucosamine and N-acetylglucosamine units. The percentage of D-glucosamine in the polymeric chain can vary from one sample to another and is expressed as the degree of deacetylation (DDA). Since this parameter has an impact on many properties, its determination is often critical, and potentiometric titration is a common analytical technique to measure the DDA. Cross-linking with glutaraldehyde is one of the most explored modifications of chitosan; however, the determination of the DDA for the resulting reticulated chitosan resins can be challenging. In this paper, we report a new, rapid, and efficient method to determine the DDA of glutaraldehyde-cross-linked chitosan resins via HPLC. This method relies on the use of 2,4-dinitrophenylhydrazine (DNPH) as a derivatizing agent to measure the level of reticulation of the polymer (LR) after the reticulation step. In this study, we prepare three calibration curves (with an *R*^2^ value over 0.92) for three series of reticulated polymers covering a large range of reticulation levels to demonstrate that a correlation can be established between the LR established via HPLC and the DDA obtained via titration. The polymers are derived from three different chitosan starting materials. These standard calibration curves are now used on a routine basis in our lab, and the HPLC method has allowed us to change our DDA analysis time from 20 h to 5 min.

## 1. Introduction

Chitosan is a natural biopolymer derived from the partial deacetylation of chitin, which is a major component of the shells of crustaceans such as shrimp, and crabs [1,2]. Chitosan is a linear biopolymer composed of randomly distributed D-glucosamine and N-acetylglucosamine units chained together by a β 1–4 glycosidic linkage. The ratio of the two monomers in the polymeric chain can vary from one sample to another and is commonly expressed as the degree of deacetylation (DDA), which corresponds to the percentage of D-glucosamine units in the polymer chains.

Due to its biocompatibility and biodegradability, chitosan has received considerable attention. Its wide range of applications include wastewater treatment, pharmaceuticals, and agriculture [3,4]. By modifying the reactive functional groups (mainly amino and hydroxyl groups) chitosan can be easily tailored to suit various applications [5,6]. 

**Figure 1:**
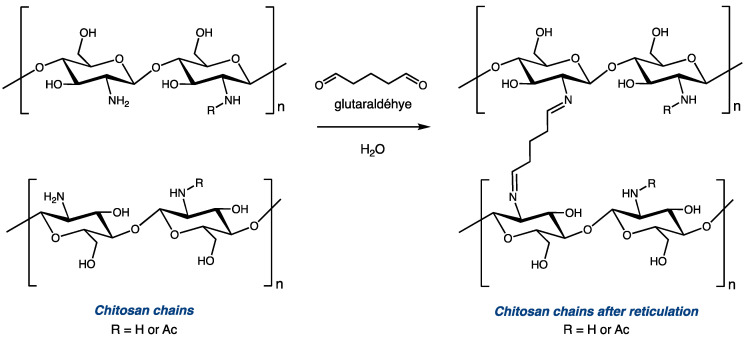
Reticulation of chitosan with glutaraldehyde (traditional representation).

Cross-linking with glutaraldehyde is one of the most explored modifications of chitosan, as it is an inexpensive and straightforward way to efficiently modulate the structure and mechanical properties of the biopolymer [7,8,9,10,11,12,13,14,15]. Glutaraldehyde, with two aldehyde functionalities, can react with two amino functionalities to covalently bond two distinct polysaccharide chains via the installation of a C5 diimide linker (Figure 1). Depending on the reaction conditions (mainly the molar ratio between the amino group of chitosan and glutaraldehyde), modified polymers with different levels of reticulation can be obtained from the same starting material. Controlling this parameter can be critical for certain applications. On the one hand, cross-linking chitosan with glutaraldehyde increases its mechanical resistance and its chemical stability [12,13,14]. On the other hand, high levels of reticulation significantly impact the overall solubility and swelling properties of chitosan derivatives [15,16]. Thus, it is important to use proper tools to accurately evaluate the extent to which the polymer chains are cross-linked after the reticulation reaction. A comparison between the DDA of chitosan before and after the modification can help to determine the ratio of glucosamine units that were used to reticulate the polymeric chains.

Currently, there are several methods to determine the DDA of unmodified or modified chitosan resins, including direct quantitative methods and indirect analytical methods such as infrared spectroscopy [17], ^1^H and ^13^C NMR [18], X-ray diffraction [19], ultraviolet spectroscopy [20], and potentiometric titration [21,22,23]. ^1^H NMR is often regarded as the modern reference method to determine the DDA [24,25]. However, this technique may not be suitable for all laboratories, as it requires costly instrumentation. Moreover, ^1^H NMR analyses of DDA can rapidly become quite challenging as modifications are made to the chitosan that complexify its structure (this is true for most of the methods listed above). Alternatively, potentiometric titration is a technique that is quite versatile and can accommodate a broad scope of substrates thanks to its simplicity; free amines are directly quantified through a protonation–deprotonation mechanism. One other advantage is that it relies on readily accessible reagents and equipment [21,22,23]. However, the main disadvantage of this method is that it can quickly become time-consuming as the number of sample increases [25], particularly for certain modified chitosan resins for which the acid–base equilibrium can be slower to achieve due to poor solubility in the titration medium or a more rigid structure that impedes access the to the basic amine functionalities of the polymer. 

Recently, while preparing a series of glutaraldehyde-cross-linked chitosan resins, it took us about 20 h per sample to obtain a reproductible titration curve. Consequently, we decided to try to develop a complementary method that would be faster and allow us to treat a larger number of samples in less time. HPLC was chosen as the technique of choice to develop this new method as HPLC works quickly and is readily available to most laboratories. To date, HPLC has been mostly used to determine the DDA of unmodified chitosan resins [26,27,28,29,30]. To the best of our knowledge, it had never yet been used with cross-linked chitosan derivatives. 

Herein, we report a new, rapid, and efficient method to determine the DDA of glutaraldehyde-cross-linked chitosan resins. This method relies on the use of 2,4-dinitrophenylhydrazine (DNPH) as a derivatizing agent to measure the amount of residual glutaraldehyde after the reticulation reaction. We show that for chitosan samples sharing the same range of degree of polymerization, a linear correlation can be established between the DDA values (obtained from potentiometric titration) and the quantification of residual glutaraldehyde via HPLC. Therefore, our new proposed method offers an alternative to potentiometric titration that is more suited for the rapid analysis of a large set of related samples. 

## 2. Results and Discussion

As we worked to develop new modified biopolymers with enhanced metal-binding properties, we were interested in preparing different chitosan derivatives with various degrees of polymerization (DP) and levels of reticulation. 

We first acquired three high-grade chitosan resins (ChitoClear^®^ HQG, Primex ehf, Siglufjörður Iceland). The information on those starting materials is summarized in Table 1. Ranges of molecular weight and viscosity values were directly given by the manufacturer, while we determined the DDA of the starting materials (DDA_SM_) using potentiometric titration. The statistical analysis showed a significant difference between the degree of deacetylation (DDA_SM_, %) of the three chitosans.

These starting materials were reticulated using the glutaraldehyde cross-linking reaction. To prepare chitosan resins with different levels of reticulation, each of the three starting materials was divided into 5 parts and each fraction was submitted to a different molar ratio of glutaraldehyde. To establish the stoichiometry of those reactions, only the monomeric units with free NH_2_ groups were considered for chitosan, so the ratio measured was that of glutaraldehyde to free amines (GLA/NH_2_ ratio). Five different ratios were explored, ranging from 1:4 to 4:1. 

To assess the impact of the glutaraldehyde concentration used on the properties of the resulting modified chitosan resins, we determined the DDA of each product via potentiometric titration (DDA_RET_). Then, the DDA values of the starting materials (DDA_SM_) and the reticulated products (DDA_RET_) could be directly compared and used to establish the level of reticulation of each modified chitosan resin as a fraction of the D-glucosamine units involved in the cross-linking of the polymer chains (LR_TITR_, in %). The results are summarized in Table 2. FTIR analyses were also performed on all modified chitosan resins and we confirmed that glutaraldehyde was successfully incorporated (see Supplementary Materials) [31,32].

The results in Table 2 show that the DDA_RET_ values do not follow a linear relationship with the molar equivalent of glutaraldehyde. For HQG 400 and 800, a certain threshold of reticulation is reached around the 2:1 (GLA/NH_2_) ratio, while the DDA remains fairly consistent when using a 4:1 ratio (Table 2, entries 9 vs. 10 and 14 vs. 15). However, for ChitoClear^®^ HQG 10, a decrease in the DDA was observed when increasing the molarity of glutaraldehyde for all tested concentrations (Table 2, entries 1–5). Our results suggest that once they reach a certain level of reticulation, the longer polymer chains of ChitoClear^®^ HQG 400 and 800 create a rigid network of cross-linked chains in which glutaraldehyde can barely penetrate to react with the remaining free amino groups. 

As shown in Table 2, using potentiometric titration allowed us to obtain reliable and reproducible DDA values for our modified chitosan resins. However, this technique was time-consuming. While the DDA of unmodified chitosan could be obtained in minutes, the same analysis for a single reticulated derivate took about 20 h. As the pH was exceedingly slow to stabilize between each titration point, we had to add NaOH (the reagent used to titrate the protonated free amine groups of chitosan) very slowly to obtain a reliable titration curve for our modified resins. 

This major difference between modified and unmodified chitosan resins is most probably explained by their respective solubility in the titration medium [33]. While the unmodified chitosan starting materials stay in solution for most of the titration process, the reticulated derivatives remain in suspension through the whole covered pH range, suggesting that the acid–base equilibrium may be slower to achieve for those latter samples. The more rigid structure of the reticulated polymers may also be a contributing factor, as cross-linking could possibly impede access to the basic amine functionalities of the polymer.

Since our group frequently synthesizes glutaraldehyde-cross-linked chitosan, we decided to try to develop a faster method to determine the DDA of a larger number of samples in a shorter timeframe. We hypothesized that 2,4-dinitrophenylhydrazine (DNPH) could be used with HPLC to quantify the residual glutaraldehyde after the reticulation reaction of chitosan, thereby allowing the indirect determination of how much aldehyde was consumed by the chitosan. DNPH is a common derivatizing agent that has been extensively used for the quantification of various carbonyl compounds [34,35,36,37,38]. Furthermore, DNPH has proven to be a useful reagent to develop analytical methods dedicated specifically to glutaraldehyde [39,40,41,42]. 

**Figure 2:**
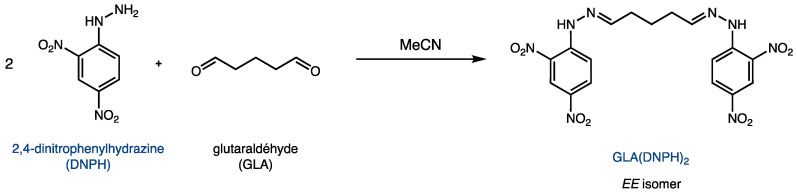
Derivatization of glutaraldehyde (GLA) with 2,4-dinitrophenylhydrazine (DNPH).

The reaction between glutaraldehyde and DNPH yields the product GLA(DNPH)_2_, as shown in Figure 2. Because of its two hydrazone functionalities, this derivative can be quantified using UV spectroscopy. Although the major product is the *EE* isomer shown in Figure 2, two other geometric isomers (*EZ* and *ZZ*) are possible due to the two C=N bonds. 

The same set of reticulation reactions discussed above (and shown in Table 2) was used to explore the development of our new DNPH-based methodology. In parallel to isolating the resulting polymers via filtration, the filtrates were conserved and derived with a DNPH solution prepared in slightly acidic acetonitrile. The preparation of a calibration curve for GLA(DNPH)_2_, followed by the analysis of our filtrate samples via HPLC, led us to quantify the residual glutaraldehyde for each reaction (see Supplementary Materials for details and for complete data). To prevent any possible detector drift, a calibration curve with fresh standards was prepared before each new set of samples. The comparison between those calibration curves (see Supplementary Materials) confirmed the reproducibility of the method. 

It is noteworthy to mention that in all our analyses, two peaks were observed for GLA(DNPH)_2_, demonstrating the presence of two geometric isomers. This phenomenon has been previously reported and the two isomers can be identified as *EE* and *EZ* [39,42,43,44]. This observation was expected in our study, since H_3_PO_4_ (approximately 0.2 M) was added in the derivatization medium to catalyze the formation of GLA(DNPH)_2_ and promote rapid equilibration between the geometric isomers. This is to ensure that the ratio between the two isomers does not change during the analysis and distort the results (the two isomers have slightly different molar absorption coefficients at the observed wavelength). As expected, the ratio between the areas of the two peaks was consistent in all of our chromatograms, confirming that the equilibrium between the two isomers was reached by the time of the HPLC analysis (see Supplementary Materials for detailed values and statistics regarding those ratios). Since this condition was met, the area of the two peaks could be added and treated as one for quantification, as demonstrated by Uchiyama et al. [40,42]. No product of monoaddition (GLA(DNPH)) resulting from an incomplete derivatization of glutaraldehyde was observed during our HPLC analysis. Two example chromatograms (with peak assignation) are shown in the Supplementary Materials. 

Once the concentrations of residual glutaraldehyde were established, we determined the amount of glutaraldehyde consumed in each reaction. This allowed us to evaluate the ratio of the free amino groups of chitosan that were involved in the cross-linking reaction (see Section 3.5.4 for data treatment and see the Supplementary Materials for a detailed example). This ratio can be expressed as the level of reticulation (LR_HPLC,_ in %)_._ Table 3 presents those results. 

The direct comparison between the values obtained for the level of reticulation (LR_TITR_ in Table 2 vs. LR_HPLC_ in Table 3) shows that the two analytical methods differ significantly. For most experiments, the LR_HPLC_ was higher than the LR_TITR_, and in general the difference between the two values increased with higher concentrations of glutaraldehyde. Most notably, when treating the starting materials with the two highest concentrations of glutaraldehyde, values of LR_HPLC_ over 100% were observed in almost all cases (Table 3, entries 4, 5, 10, 14, and 15). 

Although such high levels of incorporation of glutaraldehyde in our reticulated chitosan do not seem possible considering the traditional theoretical representation of glutaraldehyde-cross-linked chitosan (in which there is a 2:1 ratio between NH_2_ and GLA; see Figure 1), they can be rationalized by the hypothesis that glutaraldehyde can form oligomers prior to its addition to chitosan. It has been shown that, in certain reaction conditions, glutaraldehyde can be oligomerized by aldol condensation [45,46,47,48,49]. The same hypothesis was already proposed by Hsien and Rorrer to explain a higher-than-expected glutaraldehyde incorporation rate in the heterogeneous cross-linking of chitosan gel beads [11]. 

Although they can be rationalized, the LR_HPLC_ values calculated by our HPLC method obviously do not give an accurate picture of the fraction of the glucosamine units involved in cross-linking. Therefore, those values cannot be used to properly evaluate the DDA values of our chitosan resins after reticulation, and our proposed HPLC method does not offer a direct alternative to the determination of the DDA via potentiometric titration. 

**Figure 3:**
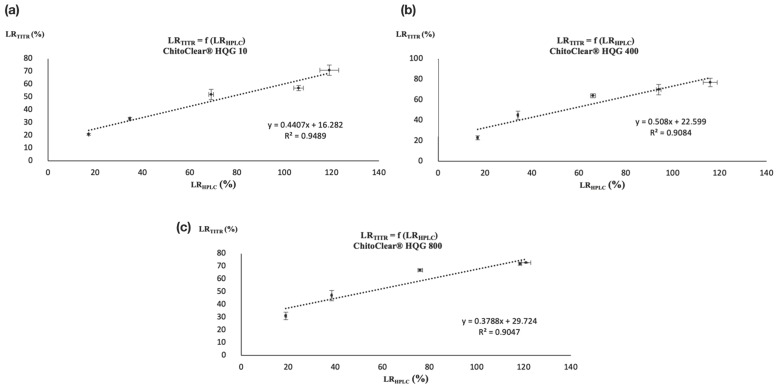
Correlation between LR_TITR_ and LR_HPLC_ for (**a**) ChitoClear^®^ HQG 10, (**b**) ChitoClear^®^ HQG 400, and (**c**) ChitoClear^®^ HQG 800.

Nevertheless, the similar trends observed between Table 2 and Table 3 suggest that a correlation may exist between the two analytical techniques. Both analytical methods showed that a comparable level of reticulation was attained for the three starting materials when treated with the highest concentration of glutaraldehyde (entries 5, 10, and 15 for Table 2 and Table 3). Moreover, as was the case for potentiometric titration, the HPLC analysis also suggests that a plateau in the level of reticulation was reached for ChitoClear^®^ HQG 800 for the two highest concentrations of glutaraldehyde used (entries 14 and 15, Table 2 and Table 3). To check if those similarities reflect the existence of a real correlation, we plotted the LR_TITR_ and LR_HPLC_ together for ChitoClear^®^ HQG 10, HQG 400, and HQG 800. As shown in Figure 3, linear correlations with *R*^2^ values over 0.90 exist for the three series of reticulated chitosan resins.

**Figure 4:**
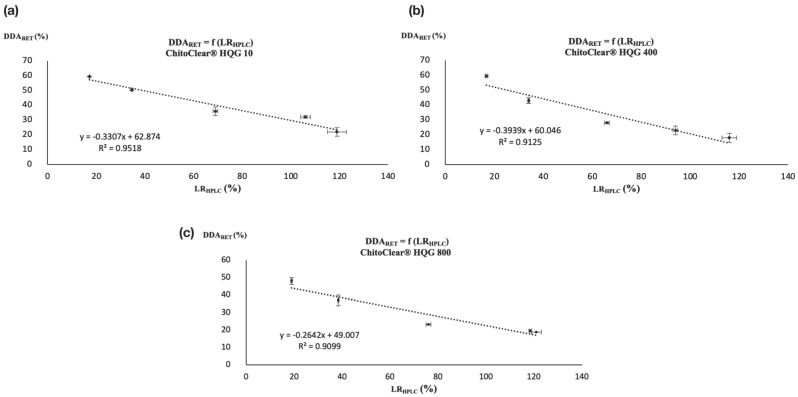
Correlation between DDA_RET_ and LR_HPLC_ for (**a**) ChitoClear^®^ HQG 10, (**b**) ChitoClear^®^ HQG 400, and (**c**) ChitoClear^®^ HQG 800.

Since for each series of reticulated chitosan resins the LR_TITR_ was calculated with the DDA of the starting material (DDA_SM_) and the DDA after reticulation was calculated via potentiometric titration (DDA_RET_), a linear correlation was also found between the LR_HPLC_ and DDA_RET_, as illustrated in Figure 4. Once again, the correlation coefficient (*R*^2^) was over 0.90.

The graphics in Figure 4 show that HPLC is a serviceable alternative to using potentiometric titration to determine the DDA of glutaraldehyde-cross-linked chitosan resins. The derivatization of residual glutaraldehyde with DNPH followed by dosage via HPLC allowed us to obtain a certain level of reticulation (LR_HPLC_), which although it does not reflect the real extent of cross-linking between the polymeric chains can be used to calculate the DDA of the modified chitosan using a simple linear relationship. In that sense, Figure 4a–c show three calibration curves that can be used to indirectly determine the DDA of glutaraldehyde-cross-linked chitosan resins prepared from HQG-10, HQG-400 and HQG-800 using Equations (1), (2) and (3), respectively:

DDA_RET [HQG-10]_ = −0.3307(LR_HPLC [HQG-10]_) + 62.874 (1)

DDA_RET [HQG-400]_ = −0.3939(LR_HPLC [HQG-400]_) + 60.046 (2)

DDA_RET [HQG-800]_ = −0.2642(LR_HPLC [HQG-800]_) + 49.007 (3)

This new HPLC methodology has been adopted by our group and is now used on a routine basis to evaluate the DDA values of new batches of glutaraldehyde derivatives of HQG-10, HQG-400, and HQG-800. Regular control experiments (comparisons of DDA obtained via interpolation with the calibration curve and DDA values obtained directly via titration) have been run and have shown that the three calibration curves remain reliable over time. 

Although it took some effort to develop, this new HPLC methodology has overall allowed us to optimize our workflow (compared to traditional potentiometric titration), as the time allowed to determine the DDA of each sample has been drastically reduced (from 20 h to 5 min). In addition to the three examples presented in this paper, this methodology could also be applied to other chitosan starting materials (from other manufacturers or with other degrees of polymerization), as long as a calibration curve is established beforehand. 

## 3. Materials and Methods

### 3.1. Materials

The three chitosan starting materials were purchased from Primex (ChitoClear^®^ HQG 10 (product code 43000TM4045), ChitoClear^®^ HQG 400 (product code 43020TM4375), ChitoClear^®^ HQG 800 (product code 43030TM5468)). All chemical reagents, including hydrochloric acid, glutaraldehyde (approximately 50% in water at 5.6 mol/L), sodium hydroxide, 2,4-dinitro-phenylhydrazine (DNPH), acetonitrile (suitable for HPLC), and phosphoric acid, were purchased from MilliporeSigma and used as purchased.

### 3.2. Determination of DDA Values of Starting Materials via Potentiometric Titration

Commercial chitosan (ChitoClear^®^ HQG 10, ChitoClear^®^ HQG 400, or ChitoClear^®^ HQG 800) samples were lyophilized prior to titration. For each starting material, a solution of 50 mg of polymer in 20 mL of diluted HCl (0.03 mol/L) was prepared and stirred for 30 min to allow sufficient time to charge all free amines. The resulting solution was then titrated with NaOH solution (0.1 mol/L). First, 3 mL of this solution was added rapidly to the sample. Then, the basic solution was added via the incremental addition of 100 μL every 30 s (total titration time of 20 min).. The volume of added NaOH and the pH values of the solution were recorded using an automatic titrator (848 Titrino Plus, Metrohm, Herisau, Switzerland). Prior to each titration, calibration of the pH electrode was performed with buffer solutions of pH 4.00 and 7.00.

Two inflection points were recorded. The first inflection point corresponded to the volume necessary to neutralize the excess HCl acid used to dissolve the chitosan and fully protonate all of its amino functionalities (*V*_1_), while the second corresponded to the complete neutralization of the medium (*V*_2_). The difference between the two points (*V*_2_–*V*_1_) was the volume of NaOH necessary to neutralize all ammonium functions of the chitosan. Three replicates were performed for each starting material. The DDA (%) was calculated using Equation (4), as proposed by Broussignac [21]:

(4) $$\mathrm{DDA}=100\frac{203.19\left(V_{2}-V_{1}\right)\left[\mathrm{NaOH}\right]}{m+42.03\left(V_{2}-V_{1}\right)\left[\mathrm{NaOH}\right]}\;$$

where [NaOH] is the concentration (0.1 mol/L) of the sodium hydroxide solution used for titration, (*V*_2_ − *V*_1_) is the volume of HCl required to neutralize the ammonium functionalities (L), 203.19 (g/mol) is the molecular weight of the N-acetyl-glucosamine unit, 42.033 (g/mol) is the difference between the molecular weight of N-acetyl-glucosamine unit and that of the D-glucosamine unit, and *m* is the mass (g) of the sample in the dry state before titration.

Each starting material was analyzed in triplicate and the final results are reported as means ± standard deviations. An example of a titration curve and a calculation of the corresponding DDA_SM_ are in the Supplementary Materials. 

### 3.3. Preparation of Glutaraldehyde-Cross-Linked Chitosan

Glutaraldehyde-cross-linked chitosan resins with different levels of reticulation were prepared from ChitoClear^®^ HQG 10, ChitoClear^®^ HQG 400, and ChitoClear^®^ HQG 800, using a procedure adapted from Hsien and Rorrer [11]. The three starting materials were divided into 5 fractions each and reacted with different concentrations of glutaraldehyde, corresponding to various glutaraldehyde/free amino group ratios (GLA/NH_2_). Ratios of 1:4, 1:2, 1:1, 2:1, and 4:1 were tested for each starting material. Each combination (starting material/GLA/NH_2_ ratio) was performed in triplicate. 

For each reaction, 2 g of lyophilized chitosan was added to 10 mL of distilled water in a conical centrifuge tube. Then, a certain volume (calculated to reach the desired GLA/NH_2_ ratio and rounded to the closest 0.1 mL) of a 5.6 mol/L glutaraldehyde solution in water was added and the volume was topped up to 40 mL with water before the tube cap was tightly screwed. The reaction was stirred for 48 h at room temperature using a benchtop laboratory shaker (SHAKER SK-71 Lab Companion at 230 rpm). The solution was then centrifuged at 3400× *g* for 6 min to separate the two phases, with the pellet corresponding to glutaraldehyde-cross-linked chitosan and the supernatant containing the unreacted glutaraldehyde. The supernatant was filtered in a syringe fitted with a 0.45 μm nylon filter and treated immediately for the analysis (see Section 3.5.3). The settled cross-linked chitosan was rinsed 5 times with a volume of 30 mL of distilled water (with a 5 min agitation period followed by centrifugation at 3400× *g* for 6 min). After the fifth rinse, the supernatant water was removed and the remaining solid was lyophilized. 

### 3.4. Potentiometric Titration of Glutaraldehyde-Cross-Linked Chitosan

The DDA for the cross-linked chitosan prepared in Section 3.3 was determined using the same procedure as detailed in Section 3.2, except that the time between each incremental addition of NaOH had to be set to 1200 s to obtain reliable titration curves (total titration time = 20 h). Once those curves were obtained, the DDA_RET_ values were obtained using Equation (4) (Section 3.2). The results are reported as means ± standard deviations.

The modified chitosan remained in suspension throughout the titration, in contrast to unmodified chitosan, which stays in solution for most of the titration process. An example of a titration curve and the determination of the corresponding DDA is provided in the Supplementary Materials.

Once the DDA_RET_ was established for each modified chitosan resin, the level of reticulation could be obtained using Equation (5):

(5) $${\mathrm{LR}}_{\mathrm{TITR}}=100\frac{{\mathrm{DDA}}_{\mathrm{SM}}-{\;\mathrm{DDA}}_{\mathrm{RET}}}{{\mathrm{DDA}}_{\mathrm{SM}}}$$

The uncertainties of those values were calculated using the upper–lower bound method of uncertainty propagation. An example of the LR_TITR_ calculation is provided in the Supplementary Materials.

### 3.5. Determination of the Level of Reticulation via HPLC (LR_HPLC_)

#### 3.5.1. Preparation of a 2,4-dinitrophenylhydrazine (DNPH) Stock Solution

In a 250 mL volumetric flask, 0.36 g (1.81 mmol) of DNPH and 30 mL of acetonitrile were added and the mixture was agitated. Then, 1.25 mL of 85% phosphoric acid was added before the volume was completed with acetonitrile. 

#### 3.5.2. Preparation of an HPLC Calibration Curve for GLA(DNPH)_2_

First, a stock solution of glutaraldehyde was prepared in a 100 mL volumetric flask by introducing 250 µL of glutaraldehyde (approximately 50% in water at 5.6 mol/L) and completing the volume with acetonitrile. Five standards were prepared from this stock solution by transferring 10, 15, 30, 50, and 200 µL to 5 mL amber volumetric flasks. In each flask, 2 mL of the DNPH stock solution (prepared as described in Section 3.5.1) was also transferred before the volumes were all completed to 5 mL with acetonitrile. The HPLC data were recorded on a Agilent Technologies 1200 Series instrument with a Restek Roc C18 column (150 mm × 4.6 mm, 5 μm) and a DAD detector (λ = 365 nm). All solvents were degassed and a mix of 25% H_2_O/75% CH_3_CN was used at a flow rate of 1.2 mL/min for 5 min. The injection volume was set to 10 µL. Under these conditions, two peaks were observed for GLA(DNPH)_2_ (4.04 min and 4.50 min), corresponding to two geometric isomers (*EE* and *EZ*). A standard ratio of 3.4 (±0.1):1 was observed between the two peaks for all standards, confirming that equilibrium in isomerization was attained during the analysis [42]. Consequently, for all standards, the integration of those two peaks was added and used as the total area. A typical calibration curve and an example of a chromatogram for a GLA(DNPH)_2_ standard can be found in the Supplementary Materials. A new calibration curve with fresh standards was constructed at the beginning of the analysis of every new set of samples.

#### 3.5.3. Determination of Residual Glutaraldehyde after Cross-Linking Reaction

The supernatant collected after each reticulation reaction (see Section 3.3) was used to quantify the remaining glutaraldehyde. Depending on the GLA/NH_2_ ratio used for the reticulation reaction, a different dilution factor was applied to fit the calibration curve. Precisely, an aliquot of 1 μL (for 4:1 ratio), 2 μL (for 2:1 ratio), 0.2 mL (for 1:1 ratio), 1 mL (for 1:2 ratio), or 2 mL (for 1:4 ratio) of the solution sampled from the supernatant was placed in an amber volumetric flask. Then, 2 mL of the DNPH stock solution (prepared as described in Section 3.5.1) was added to each vial. Finally, an adequate amount of acetonitrile was added to each vial until a final volume of 5 mL was reached. Those solutions were analyzed via HPLC (immediately after their preparation) using the same method as detailed in Section 3.5.2. The concentrations of GLA(DNPH)_2_ were interpolated using a calibration curve (prepared as described in Section 3.5.2), giving the residual glutaraldehyde concentration as [GLA_res_HPLC_] (mol/L).

#### 3.5.4. Data Treatment

The level of reticulation determined via HPLC (LR_HPLC_) is defined by Equation (6):

(6) $${\mathrm{LR}}_{\mathrm{HPLC}}=100\;\frac{n\;\left({\mathrm{NH}}_{2\_\mathrm{reacted}}\right)}{n\left({\mathrm{NH}}_{2\_\mathrm{SM}}\right)}$$

where *n*(NH_2_reacted_) is the quantity (mol) of free amino groups of the starting material that were involved in the cross-linking reaction and *n*(NH_2_SM_) is the quantity (mol) of free amino groups available in the sample of the starting material before the cross-linking reaction.

The terms *n*(NH_2_reacted_) and *n*(NH_2_SM_) can be developed to include [GLA_res_HPLC_] (introduced in Section 3.5.3) and other measurable parameters to yield Equation (7):

(7) $${\mathrm{LR}}_{\mathrm{HPLC}}=\left(\frac{100}{2}\right)\frac{n\;\left({\mathrm{GLA}}_{\mathrm{added}}\right)-\left\{\left[{\mathrm{GLA}}_{\mathrm{res}\_\mathrm{HPLC}}\right]\cdot \mathrm{DF}\cdot V_{rxn}\right\}}{n\left(\mathrm{monomer}\right)\;\frac{{\mathrm{DDA}}_{\mathrm{SM}}}{100}\;}$$

where, *n*(GLA_added_) is the initial quantity of glutaraldehyde added to the reaction mixture (mol), [GLA_res_HPLC_] is the residual glutaraldehyde concentration as determined via HPLC (mol/L), DF is the dilution factor between the crude reaction medium and the sample analyzed via HPLC, *V_rxn_* is the total volume of the reticulation reaction medium (L), *n*(monomer) is the total amount of monomers (mol) in the chitosan starting material sample submitted to cross-linking, and DDA_SM_ is the degree of deacetylation of that same starting material.

The term *n*(monomer) can be developed using Equation (8):

(8) $$n\left(\mathrm{monomer}\right)=\frac{m\left(\mathrm{sample}\right)}{M_{av\_mono}}$$

Here, *m*(sample) is the mass of chitosan starting material submitted to the reticulation reaction (g) and *M_av_mono_* is the average molar mass of the monomer of that same starting material (g/mol). 

The average molar masses of the monomer units (*M_av_mono_*, in g/mol) for the three chitosan starting materials were 171.67 ± 0.08, 170.6 ± 0.3, and 174.02 ± 0.04 for ChitoClear^®^ HQG 10, HQG 400 and HQG 800, respectively. Those values were calculated using Equation (9):

(9) $$M_{av\_mono}=\frac{{\mathrm{DDA}}_{\mathrm{SM}}}{100}\cdot \;M_{glucosamine}+\frac{\left(100-{\mathrm{DDA}}_{\mathrm{SM}}\right)}{100}M_{N-acetylglucosamine}$$

Here, DDA_SM_ is the degree of deacetylation of the chitosan starting material, *M_glucosamine_* is the molar mass of a glucosamine monomer unit (161.16 g/mol), and *M_N-acetylglucosamine_* is the molar mass of a N-acetylglucosamine monomer unit (203.19 g/mol). Details on the calculation of *M_av_mono_* can be found in the Supplementary Materials.

Finally, substituting Equation (8) into Equation (7) leads to Equation (10), which can be directly used to determine the LR_HPLC_ from a HPLC trace.

(10) $${\mathrm{LR}}_{\mathrm{HPLC}}=\left(\frac{100\;}{2}\right)\frac{n\;\left({\mathrm{GLA}}_{\mathrm{added}}\right)-\left\{\left[{\mathrm{GLA}}_{\mathrm{res}\_\mathrm{HPLC}}\right]\cdot \mathrm{DF}\cdot V_{reaction}\right\}}{\left[\frac{m\left(\mathrm{sample}\right)}{M_{av\_mono}}\right]\frac{{\mathrm{DDA}}_{\mathrm{SM}}}{100}\;}$$

The uncertainties of the LR_HPLC_ values were calculated by considering the uncertainties of the DDA_SM_ and *M_av_mono_* using the upper–lower bound method of uncertainty propagation.

The Supplementary Materials show the complete equation treatment (from Equation (6) to Equation (10)) and a complete detailed example.

### 3.6. Statistical Analysis

All analyses were performed in triplicate, unless otherwise indicated. The data are expressed as means ± standard deviations (SD) or mean ± the uncertainty provided using the upper–lower bound method of uncertainty propagation (see Supplementary Materials for detailed examples of our calculations). Statistical analyses were performed using a one-way analysis of variance with a post hoc Tukey’s honest significant difference test (Tukey’s HSD test). All results were considered statistically significant at *p* < 0.001.

## 4. Conclusions

We developed a new HPLC method to determine the degree of deacetylation (DDA) for glutaraldehyde (GLA)-cross-linked chitosan resins. Our method relies on the derivatization of residual GLA with DNPH, then quantifies the GLA(DNPH)_2_ adduct via HPLC. This method allowed us to determine the quantity of GLA incorporated into the biopolymer for three sets of reactions involving three chitosan starting materials with different polymeric chain lengths (ChitoClear^®^ HQG 10, HQG 400, and HQG 800) and various equivalents of GLA. Interestingly, the values obtained showed that in some cases more equivalents of GLA are consumed than the traditional GLA–chitosan cross-linking model allows, suggesting that oligomerization of GLA occurs before its reaction with chitosan. Due to this particular behavior of GLA, the developed method did not allow us to directly measure the DDA values via HPLC. However, for the three sets of reactions, a correlation could be established between the quantities of GLA(DNPH)_2_ dosed in the reaction medium and the DDA values of the resulting modified chitosan resins obtained via potentiometric titration. In both cases, a linear correlation with an *R*^2^ value of more than 0.90 was observed between the level of reticulation (as calculated with the traditional GLA–chitosan cross-linking model) obtained via HPLC and the DDA obtained via titration. These calibration curves were adopted by our lab to evaluate the DDA values of samples of GLA-cross-linked ChitoClear^®^ HQG 10, HQG 400, and HQG 800 on a routine basis using HPLC. This methodology allowed us to use rapid HPLC runs (5 min) instead of time-consuming titrations (20 h), thereby significantly optimizing our workflow. In the near future, we intend to use this new method to explore the cross-linking of chitosan resins with a more diverse array of degrees of polymerization.

## Figures and Tables

**Table 1:** Information on the length of polymer chains (expressed by viscosity and molecular weight (MW)) and degree of deacetylation (DDA_SM_, %) for the three chitosan starting materials used in this study.

Commercial Name	Viscosity (cps) ^I^	Calculated MW (KDa) ^I^	DDA_SM_ (%) ^II^
ChitoClear^®^ HQG 10	<20	110–150	75.0 ± 0.2 ^a^
ChitoClear^®^ HQG 400	200–600	250–300	77.5 ± 0.6 ^b^
ChitoClear^®^ HQG 800	600–1200	300–340	69.4 ± 0.1 ^c^

^I^: Data provided by the manufacturer. ^II^: Determined via potentiometric titration (means and standard deviations of triplicate). Note: a, b, c = significantly different groups (Tukey’s HSD test, *p* < 0.001)

**Table 2:** Degree of deacetylation (DDA_RET_, %) and level or reticulation (LR_TITR_, %) of three chitosan starting materials after reticulation with various ratios of glutaraldehyde to free amino groups of chitosan (GLA/NH_2_).

Entry	Starting Material	GLA/NH_2_ Ratio	DDA_RET_ (%) ^I^	LR_TITR_ (%) ^II^
1	ChitoClear^®^ HQG 10	1:4	59.4 ± 0.3 ^a^	20.8 ± 0.7
2		1:2	50.5 ± 0.6 ^b,^*	33 ± 1
3		1:1	36 ± 3 ^c,^*	52 ± 4
4		2:1	32.1 ± 0.9 ^c,^*	57 ± 2
5		4:1	22 ± 3 ^d^	71 ± 4
6	ChitoClear^®^ HQG 400	1:4	59.5 ± 0.7 ^a^	23 ± 2
7		1:2	43 ± 2 ^b,^*	45 ± 4
8		1:1	28.0 ± 0.3 ^c,^*	64 ± 2
9		2:1	23 ± 2 ^d^	70 ± 5
10		4:1	18 ± 3 ^d^	77 ± 4
11	ChitoClear^®^ HQG 800	1:4	48 ± 2 ^III, a^ *	31 ± 3
12		1:2	37 ± 3 ^b,^*	47 ± 4
13		1:1	23.2 ± 0.2 ^c,^*	67 ± 1
14		2:1	19.5 ± 0.6 ^c^	72 ± 1
15		4:1	18.87 ± 0.02 ^c^	72.8 ± 0.3

^I^: Determined via potentiometric titration (means and standard deviations of triplicates, unless indicated). ^II^: Calculated using DDA_SM_ (see Table 1) and DDA_RET_. ^III^: Means and standard deviation of duplicates. Note: a, b, c, d = significantly different groups when comparing between the different ratios for one chitosan starting material (e.g., Entry 1 vs. 2–5). Tukey’s HSD test, *p* < 0.001. * Significantly different groups when comparing the same ratios between the three chitosan starting materials (e.g., entry 1 vs. entry 6 and 11). Tukey’s HSD test, *p* < 0.001.

**Table 3:** Levels of reticulation determined via HPLC (LR_HPLC_ %) for three chitosan starting materials after reticulation under various ratios of glutaraldehyde to free amino groups of chitosan (GLA/NH_2_).

Entry	Starting Material	GLA/NH_2_ Ratio	LR_HPLC_ (%)
1	ChitoClear^®^ HQG 10	1:4	17.2 ± 0.1 ^a^
2		1:2	34.6 ± 0.3 ^b^
3		1:1	69 ± 1 ^c^
4		2:1	106 ± 2 ^d,^*
5		4:1	119 ± 4 ^e^
6	ChitoClear^®^ HQG 400	1:4	16.8 ± 0.3 ^a^
7		1:2	34.0 ± 0.3 ^b^
8		1:1	66 ± 1 ^c^
9		2:1	94 ± 1 ^d,^*
10		4:1	116 ± 3 ^e^
11	ChitoClear^®^ HQG 800	1:4	18.9 ± 0.3 ^a,^*
12		1:2	38.4 ± 0.2 ^b,^*
13		1:1	76 ± 1 ^c,^*
14		2:1	118.4 ± 0.4 ^d,^*
15		4:1	121 ± 2 ^d^

Note: a, b, c, d, e = Significantly different groups when comparing between the different ratios for one chitosan starting material (e.g., Entry 1 vs. 2–5). Tukey’s HSD test, *p* < 0.001. *: Significantly different groups when comparing the same ratios between the three chitosan starting materials (e.g., entry 1 vs. entries 6 and 11). Tukey’s HSD test, *p* < 0.001.

## Data Availability

The data presented in this study are available in the Supplementary Materials.

## Supplementary Materials

The following supporting information can be downloaded at: https://www.mdpi.com/article/10.3390/molecules28217294/s1, 1. Determination of the degree of deacetylation and level of reticulation via potentiometric titration: (a) general principle; (b) determination of DDA_SM_—complete raw values; (c) determination of DDA_SM_ (example); (d) determination of average molar mass of monomer; (e) determination of DDA_RET_ and LR_TITR_—complete raw values; (f) determination of DDARET (example). 2. Determination of the level of reticulation via potentiometric titration (example). 3. Preparation of a standard curve for GLA(DNPH)2. 4. Determination of the level of reticulation via HPLC: (a) equation treatment; (b) detailed example; (c) complete data; (d) example of chromatogram. 5. FTIR spectra of chitosan resins.
