# Supplementary material for: A New and Rapid HPLC Method to Determine the Degree of Deacetylation of Glutaraldehyde-Cross-Linked Chitosan
Source: Molecules. 2023 Oct 27;28(21):7294. doi: 10.3390/molecules28217294 (PMC10647662; doi:10.3390/molecules28217294)
Supplement: Supplementary file 1 [file molecules-28-07294-s001.zip › molecules-2668818-supplementary.pdf]

Supplementary materials

A new and rapid HPLC method to determine the degree of deacetylation of glutaraldehyde-cross-linked chitosan

Ons Amamou[1,2], Jean-Philippe Denis[1], Élise Heinen[1], Taoufik Boubaker[2], and Sébastien Cardinal [1,*]

[1] Département de Biologie, Chimie et Géographie, Université du Québec à Rimouski, Rimouski, QC G5L 3A1, Canada
[2] Laboratoire de Chimie Hétérocyclique, Produits Naturels et Réactivité (LR11S39), Faculté des Sciences, Université de Monastir, Monastir, Tunisia
* Correspondence : sebastien_cardinal@uqar.ca;

__Content__

1. Determination of the degree of deacetylation and level of reticulation via potentiometric titration…2

a) General principle……2

b) Determination of DDA$_{SM}$—complete raw values……2

c) Determination of DDA$_{SM}$ (example)……3

d) Determination of average molar mass of monomer……4

e) Determination of DDA$_{RET}$ and LR$_{TITR}$—complete raw values……5

f) Determination of DDA$_{RET}$ (example)……6

2. Determination of the level of reticulation via potentiometric titration (example)……8

3. Preparation of a standard curve for GLA(DNPH)$_2$……9

4. Determination of the level of reticulation by HPLC……11

a) Equation treatment……11

b) Detailled example……13

c) Complete data……16

d) Example of chromatogram……21

5. FTIR spectra of chitosan resins……22

1. Determination of the degree of deacetylation and level of reticulation via potentiometric titration

a. General principle

The degree of deacetylation (DDA) was determined by pH metric titration for unmodified (DDA_{SM}) and modified chitosan (DDA_{RET}), using an automatic titrator (848 Titrino plus, Metrohm). In brief, chitosan is initially dissolved in HCl to protonate all the amine functionalities. Then, this solution is titrated with NaOH. The volume of sodium hydroxide used corresponds both to the volume necessary to neutralize the excess HCl acid used for dissolving the chitosan (V_1) and to neutralize the total acidity of the medium (V_2). The difference between V_2 and V_1 gives the quantity of NaOH necessary for the neutralization of the ammonium functions of the unmodified chitosan (Figure 1) and modified chitosan (Figure 2). The DDA was calculated following the equation given by Broussignac (ref. 21 of the main manuscript) [Equation (S1)]:

$$\mathrm{DDA} = 100\frac{203.19(V_2-V_1)[NaOH]}{m+42.03(V_2-V_1)[NaOH]} \quad \text{(S1)}$$

for which [NaOH] is the concentration (0.1 mol/L) of the sodium hydroxide solution used for titration, (V_2-V_1) is the volume of HCl required to neutralize the ammonium functionalities (L), 203.19 (g/mol) is the molecular weight of the N-acetyl-glucosamine unit, 42.033 (g/mol) is the difference between the molecular weight of N-acetyl-glucosamine unit and that of D-glucosamine unit, and *m* is the mass (g) of the sample in the dry state before titration.

In the case of the present study, titration was performed in triplicate for each chitosan starting material and the values were reported as means ± standard deviations.

b. Determination of DDA_{SM}—complete raw values

Table S1 Determination of DDA_{SM} : complete raw values

Entry	Starting material	Mass used for titration (g)	$[NaOH]_{(aq)}$	V_1 (mL)	V_2 (mL)	DDA_{SM} (%) as calculated with Eq (1)	DDA_{SM} (%) Mean± standard deviations
1	Chito-Clear® HQG 10	0.0503	0.1	3.866	6.066	75.069	75.0 ± 0.2
2		0.0506	0.1	3.875	6.092	75.180	
3		0.0496	0.1	3.925	6.084	74.765	
4	Chito-Clear® HQG 400	0.0496	0.1	3.844	6.073	76.804	77.5 ± 0.6
5		0.0502	0.1	3.878	6.175	77.977	
6		0.0498	0.1	3.819	6.086	77.640	
7	Chito-Clear® HQG 800	0.0497	0.1	4.068	6.056	69.578	69.4 ± 0.1
8		0.0504	0.1	4.056	6.066	69.400	
9		0.0503	0.1	4.053	6.056	69.311	

c. Determination of DDA_{SM} (example)

Figure S1 presents a titration curve for ChitoClear® HQG 400 (Table S1, Entry 4). Total titration time = 20 minutes.

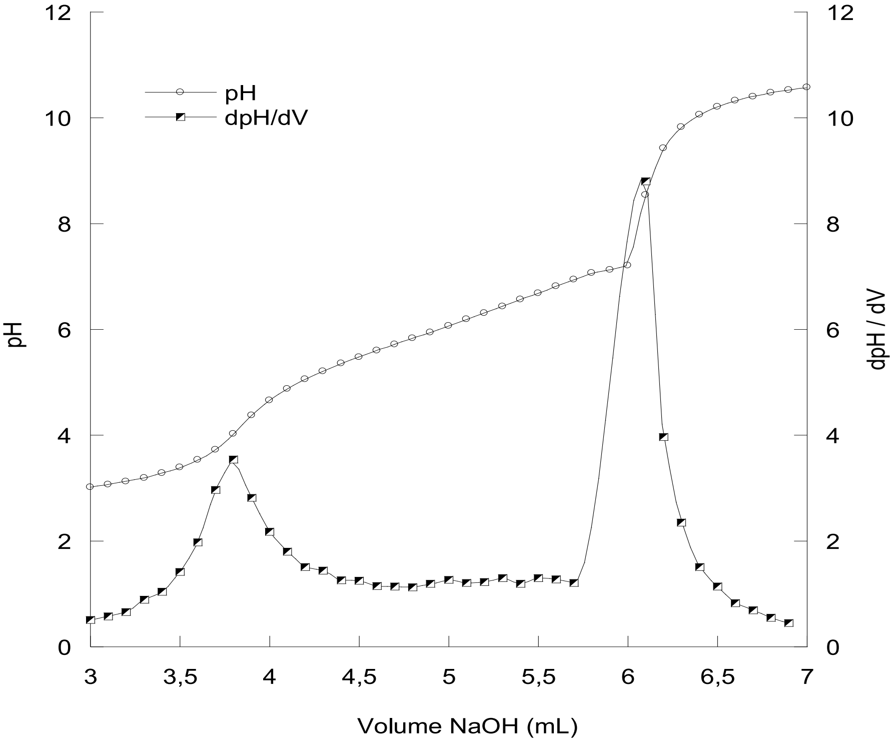

Figure S1. Potentiometric determination of DDA in starting material ChitoClear® HQG 400 (Table S1, Entry 4)

From this titration curve, V_1 and V_2 values were determinated : V_1 = 3.844 mL ; V_2 = 6.073 mL.
From there, Equation (S1) was then used to determine DDA value

$$DDA_{SM} = 100\frac{203.19(V_2-V_1)[NaOH]}{m+42.03(V_2-V_1)[NaOH]}$$

$$= 100\ \frac{203.19\ g \bullet mol^{-1} \bullet (6.073 \bullet 10^{-3}\ L - 3.844 \bullet 10^{-3}\ L) \bullet 0.1\ mol \bullet L^{-1}}{0.0496\ g + 42.033\ g \bullet mol^{-1} \bullet (6.073 \bullet 10^{-3}\ L - 3.844 \bullet 10^{-3}\ L) \bullet 0.1\ mol \bullet L^{-1}}$$

$$= 76.805$$

d. Determination of average molar mass of monomer

Once DDA_{SM} has been established, the average molar mass of a monomer unit (M_{av_mono}) can be determined for each starting material, using Equation (S2):

$$M_{av\_mono} = \frac{DDA_{SM}}{100} \cdot M_{glucosamine} + \frac{(100-DDA_{SM})}{100} M_{N-acetylglucosamine} \quad \text{(S2)}$$

for which DDA_{SM} is the degree of deacetylation of the chitosan starting material, $M_{glucosamine}$ is the molar mass of a glucosamine monomer unit (161.16 g/mol) and $M_{N\text{-}acetylglucosamine}$ is the molar mass of a N-acétyl-glucosamine monomer unit (203.19 g/mol).

For example, for ChitoClear® HQG 10, DDA_{SM} = 75.0 ± 0.2
Consequently, when applying Equation (2), we obtained

$$M_{av\_mono} = \frac{75}{100} \cdot 161.16 g \cdot mol^{-1} + \frac{(100-75)}{100} 203.19\ g \cdot mol^{-1} = 171.67\ g \cdot mol^{-1}$$

Uncertainties on this value was calculated using a variant of the upper-lower bound method of uncertainty propagation. In this case, since the two ratios of the monomer must equal 1, we used DDA_{SM} = 75.0 - 0.2 for determining $M_{av\_mono\_Max}$ and DDA_{SM} = 75.0 + 0.2 for determining $M_{av\_mono\_Min}$.

$$M_{av\_mono\_Max} = \frac{75-0.2}{100} \cdot 161.16 g \cdot mol^{-1} + \frac{(100-(75-0.2))}{100} 203.19\ g \cdot mol^{-1} = 171.75\ g \cdot mol^{-1}$$

$$M_{av_{mono\_Min}} - M_{av\_mono} = 171.75 - 171.67 = 0.08$$

$$M_{av\_mono\_Min} = \frac{75+0.2}{100} \cdot 161.16 g \cdot mol^{-1} + \frac{(100-(75+0.2))}{100} 203.19\ g \cdot mol^{-1} = 171.58\ g \cdot mol^{-1}$$

$$M_{av\_mon} - M_{av\_mono\_Min} = 171.67 - 171.58 = 0.08$$

Thus, for ChitoClear® HQG 10, final value of M_{av_mono} was reported as:

$$M_{av\_mono} = 171.67 \pm 0.08\ g \cdot mol^{-1}$$

Table S2 shows the average molar masses of monomer units (M_{av_mono}) that were calculated for each starting material, using the approach described above.

Table S2 Values calculated for $\mathbf{M_{av\_mono}}$ for chitosan starting materials.

Entry	Starting material	DDA_{SM} (%)	M_{av_mono} (g/mol)
1	ChitoClear® HQG 10	75.0 ± 0.2	171.67 ± 0.08
2	ChitoClear® HQG 400	77.5 ± 0.6	170.6 ± 0.3
3	ChitoClear® HQG 800	69.4 ± 0.1	174.02 ± 0.04

e. Determination of DDA_{RET} and LR_{TITR}—complete raw values

Table S3 Determination of DDA_{RET} for ChitoClear® HQG 10: complete raw values.

Entry	GLA/NH_2 ratio	Mass used for titration (g)	$[NaOH]_{(aq)}$	V_1	V_2	DDA_{RET} (%) as calculated with Eq (1)	DDA_{RET} (%) Mean± standard deviations	LR_{TITR} (%)
1	1 : 4	0.0500	0.1	4.375	6.049	59.636	59.4 ± 0.3	20.8 ± 0.7
2		0.0500	0.1	4.368	6.040	59.573		
3		0.0504	0.1	4.381	6.052	59.127		
4	1 : 2	0.0502	0.1	4.592	6.005	51.142	50.5 ± 0.6	33 ± 1
5		0.0503	0.1	4.564	5.950	50.177		
6		0.0510	0.1	4.573	5.978	50.168		
7	1 : 1	0.0505	0.1	4.833	5.911	39.803	36 ± 3	52 ± 4
8		0.0510	0.1	5.044	5.957	33.829		
9		0.0504	0.1	5.009	5.943	34.934		
10	2 : 1	0.0503	0.1	5.097	5.964	32.657	32.1 ± 0.9	57 ± 2
11		0.0504	0.1	5.083	5.939	32.211		
12		0.0504	0.1	5.121	5.942	30.978		
13	4 : 1	0.0498	0.1	5.442	5.959	20.212	22 ± 3	71 ± 4
14		0.0505	0.1	5.423	5.939	19.907		
15		0.0502	0.1	5.290	5.938	24.879		

Table S4 Determination of DDA_{RET} for ChitoClear® HQG 400: complete raw values.

Entry	GLA: NH_2 ratio	Mass used for titration (g)	$[NaOH]_{(aq)}$	V_1	V_2	DDA_{RET} (%) as calculated with Eq (1)	DDA_{RET} (%) Mean± standard deviations	LR_{TITR} (%)
1	1 : 4	0.0506	0.1	4.272	5.978	60.003	59.5 ± 0.7	23 ± 2
2		0.0500	0.1	4.306	5.950	58.697		
3		0.0504	0.1	4.260	5.950	59.716		
4	1 : 2	0.0500	0.1	4.692	5.918	45.167	43 ± 2	45 ± 4
5		0.0499	0.1	4.626	5.760	42.150		
6		0.0503	0.1	4.755	5.898	42.147		
7	1 : 1	0.0503	0.1	5.100	5.832	27.865	28.0 ± 0.3	64 ± 2
8		0.0508	0.1	5.105	5.840	27.713		
9		0.0502	0.1	5.088	5.831	28.312		
10	2 : 1	0.0502	0.1	5.166	5.833	25.570	23 ± 3	70 ± 5
11		0.0497	0.1	5.148	5.646	19.537		
12		0.0499	0.1	5.180	5.768	22.813		
13	4 : 1	0.0503	0.1	5.191	5.726	20.687	18 ± 3	77 ± 5
14		0.0504	0.1	5.122	5.574	17.561		
15		0.0502	0.1	5.202	5.598	15.514		

Table S5 Determination of DDA_{RET} for ChitoClear® HQG 800: complete raw values.

Entry	GLA/NH_2 ratio	Mass used for titration (g)	$[NaOH]_{(aq)}$	V_1	V_2	DDA_{RET} (%) as calculated with Eq (1)	DDA_{RET} (%) Mean± standard deviations	LR_{TITR} (%)
2		0.0494	0.1	4.616	5.861	46.304	48 ± 2	31 ± 3
3		0.0502	0.1	4.570	5.942	49.811		
4	1 : 2	0.0500	0.1	4.820	5.827	37.729	37 ± 3	47 ± 4
5		0.0500	0.1	4.790	5.855	39.723		
6		0.0503	0.1	4.850	5.751	33.848		
7	1 : 1	0.0493	0.1	5.170	5.767	23.414	23.2 ± 0.2	67 ± 1
8		0.0499	0.1	5.147	5.749	23.330		
9		0.0500	0.1	5.170	5.766	23.065		
10	2 : 1	0.0496	0.1	5.250	5.747	19.537	19.5 ± 0.6	72 ± 1
11		0.0502	0.1	5.250	5.770	20.169		
12		0.0497	0.1	5.350	5.831	18.896		
13	4 : 1	0.0498	0.1	5.350	5.831	18.860	18.87 ± 0.02	72.8 ± 0.3
14		0.0497	0.1	5.350	5.831	18.896		
15		0.0498	0.1	5.350	5.831	18.860		

f. Determination of DDA_{RET} (example)

Figure S2 shows a titration curve for reticulated ChitoClear® HQG 400 (Table S4, Entry1). Total titration time = 20 h.

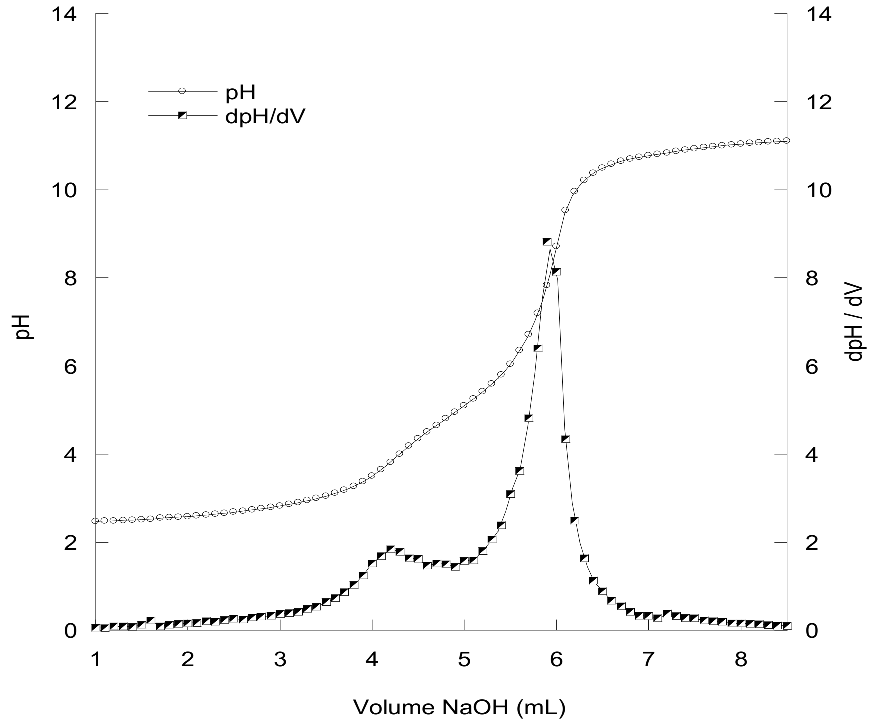

Figure S2. Potentiometric determination of *DDA* for cross-linked (GLA/NH_2 1:4) chitosan ChitoClear® HQG 400 (Table S4, Entry1)

From this titration curve, V_1 and V_2 values were determined: V_1 = 4.272 mL ; V_2 = 5.978 mL.

From there, Equation (S1) was then used to determine the degree of deacetylation.

$$DDA_{RET} = 100\frac{203.19(V_2-V_1)[NaOH]}{m+42.03(V_2-V_1)[NaOH]} \quad \text{(S1)}$$

$$= 100\,\frac{203.19\ g \bullet mol^{-1} \bullet (5.978 \bullet 10^{-3}\ L - 4.272 \bullet 10^{-3}\ L) \bullet 0.1\ mol \bullet L^{-1}}{0.0506\ g + 42.033\ g \bullet mol^{-1} \bullet (5.978 \bullet 10^{-3}\ L - 4.272 \bullet 10^{-3}\ L) \bullet 0.1\ mol \bullet L^{-1}}$$

$$= 60.003$$

2. Determination of the level of reticulation via potentiometric titration (example)

Level of reticulation determined by titration (LR_{TITR}) is defined by Equation (S3):

$$LR_{TITR} = 100\frac{DDA_{SM} - DDA_{RET}}{DDA_{SM}} \quad \text{(S3)}$$

for which DDA_{SM} is the degree of deacetylation of chitosan starting material, DDA_{RET} is the degree of deacetylation of reticulated chitosan as determined by titration.

For example, for ChitoClear® HQG 800 / GLA/NH_2 1:4 :

DDA_{RET} = 48 ± 2 (Table S5, Entries 1-3) :
DDA_{SM} = 69.4 ± 0.1 (Table S1 Entries 7-9)

$$LR_{TITR} = 100\frac{69.4-48}{69.4} = 30.84$$

Uncertainties on those values were calculated using the upper-lower bound method of uncertainty propagation.

$$LR_{TITR\_Max} = 100\frac{(69.4+0.1)-(48-2)}{(69.4-0.1)} = 33.91 \qquad LR_{TITR\_Max} - LR_{TITR} = 33.91 - 30.84 = 3.07$$

$$LR_{TITR\_Min} = 100\frac{(69.4-0.1)-(48+2)}{(69.4+0.1)} = 27.76 \qquad LR_{TITR} - LR_{TITR_{Min}} = 30.84 - 27.76 = 3.08$$

This gives a final level of reticulation determined by titration of:

$LR_{TITR} = 31 \pm 3$

3. Preparation of a standard curve for GLA(DNPH)2

Figure S3 shows a typical chromatogram obtain for a GLA(DNPH)2 standard.

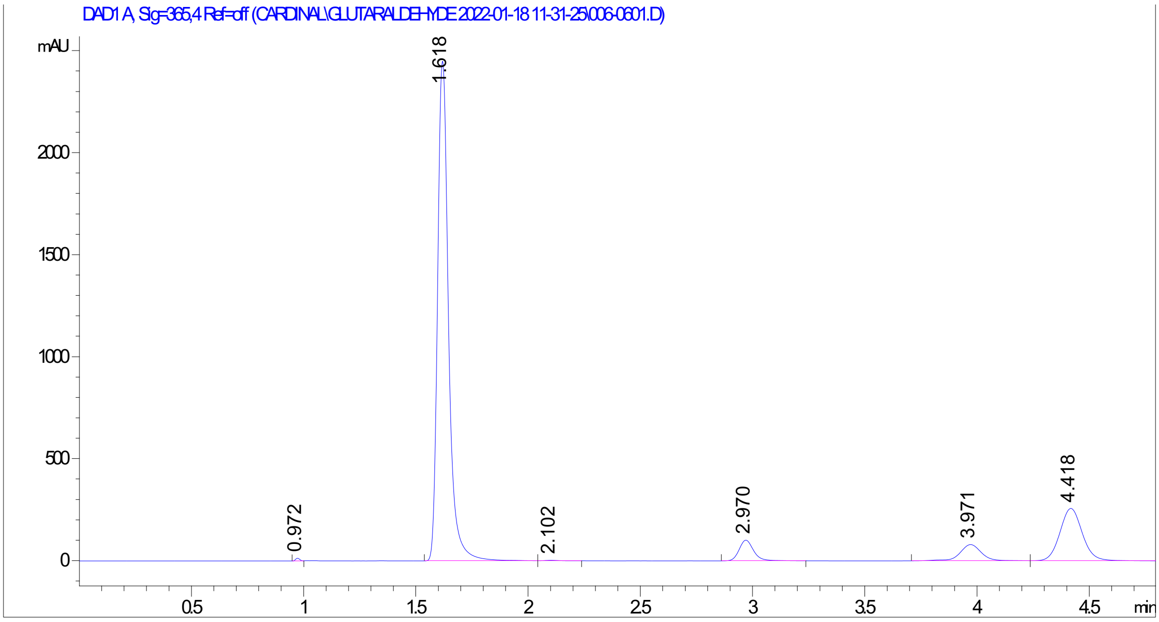

Figure S3. Chromatogram for a standard of GLA(NDNPH)2 (Table S6, Entry 5). Peaks at 3.971 min and 4.418 min correspond to GLA(DNPH)2. Peak at 1.618 min is attributed to unreacted excess DNPH. Peak at 2.970 min corresponds to an impurity that was found in the DNPH reagent. Total run time = 5 min.

A total of six standards with concentrations ranging from 1.4×10^{-5} to 5.6×10^{-4} mol/L were used to build the calibration curve. Table S6 and Figure S4 (next page) show a complete calibration curve.

Table S6 Retention time and peak area for various GLA(NDNPH)$_2$ standards

Entry	Concentration (mol/L)	Peaks retention time (min)	Peak Area	Ratio between peak area	Sum of Areas
1	1.4 10^{-5}	3.954	48	3.5	216.4
		4.399	168.4		
2	2.8 10^{-5}	4.002	97.2	3,4	427.0
		4.455	329.8		
3	4.2 10^{-5}	4.009	150.6	3,4	660.7
		4.465	510.1		
4	8.4 10^{-5}	4.014	319.6	3,4	1392.5
		4.471	1072.9		
5	1.4 10^{-4}	3.971	530.1	3,4	2306.9
		4.418	1776.8		
6	5.6 10^{-4}	4.022	2174.4	3,3	9439.0
		4.481	7264.6		

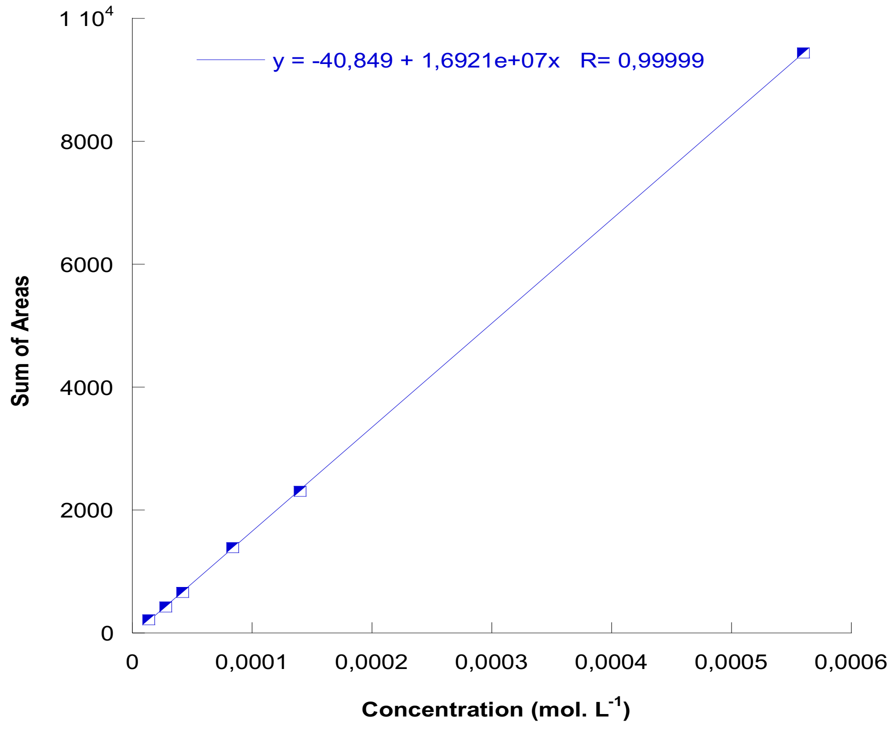

Figure S4. Calibration curve for GLA(NDNPH)$_2$

4. Determination of the level of reticulation by HPLC

a. Equation treatment

The level of reticulation determined by HPLC (LR_{HPLC}) is defined by Equation (S4):

$$LR_{HPLC} = 100\ \frac{n\ (NH_{2\_reacted})}{n(NH_{2\_SM})} \quad \text{(S4)}$$

for which $n(NH_{2\_reacted})$ is the quantity (mol) of free amino groups of the starting material that were involved in the cross-linking reaction and $n(NH_{2\_SM})$ is the quantity (mol) of free amino groups available in the sample of the starting material before the cross-linking reaction.

Considering the traditional theoretical representation of glutaraldehyde cross-linked chitosan, in which there is a 2:1 ratio between NH_2 and GLA (see Figure 1 of the main manuscript), the term $n(NH_{2\_reacted})$, can be developed as illustrated by Equation (S5):

$$LR_{HPLC} = 100\ \frac{\frac{n\ (GLA_{cons})}{2}}{n(NH_{2\_SM})} \quad \text{(S5)}$$

for which $n(GLA_{cons})$ is the quantity (mol) of glutaraldehyde consumed during the reticulation reaction.

Considering that $n(GLA_{cons})$ is the difference between the initial quantity of glutaraldehyde added to the reaction mixture and the residual quantity after the reaction, $n(GLA_{cons})$ can be developed further as illustrated by Equation (S6):

$$LR_{HPLC} = \left(\frac{100}{2}\right) \frac{n\ (GLA_{added}) - n\ (GLA_{res})}{n(NH_{2\_SM})} \quad \text{(S6)}$$

for which $n(GLA_{added})$ is the initial quantity of glutaraldehyde added to reaction mixture (mol) and $n(GLA_{res})$ is the residual quantity remaining in the reaction medium after cross-linking.

As described in Section 3.5.3, our HPLC methodology allows us to determine [GLA_{res_HPLC}] (mol/L). This value is related to $n(GLA_{res})$ by Equation S7:

$$n\ (GLA_{res}) = \left[GLA_{res\_HPLC}\right] \cdot DF \cdot V_{rxn} \quad \text{(S7)}$$

for which V_{rxn} is the total volume of the reticulation reaction medium (L) and DF is the dilution factor between the crude reaction medium and the sample analyzed with HPLC.

Merging Equation (S6) with Equation (S7) yields Equation (S8):

$$LR_{HPLC} = \left(\frac{100}{2}\right)\frac{n\ (GLA_{added}) - \{[GLA_{res\_HPLC}]DF \cdot V_{rxn}\}}{n(NH_{2\_SM})} \quad \text{(S8)}$$

The term $n(NH_{2\_SM})$ can be defined by Equation (S9):

$$n(NH_{2\_SM}) = n(monomer)\ \frac{DDA_{SM}}{100} \quad \text{(S9)}$$

in which n(monomer) is the total amount of monomer (mol) in the chitosan starting material sample submitted to cross-linking, and DDA_{SM} is the degree of deacetylation of that same starting material.

Merging Equation (S8) with Equation (S9) yields Equation (S10):

$$LR_{HPLC} = \left(\frac{100}{2}\right)\frac{n\ (GLA_{added}) - \{[GLA_{res\_HPLC}] \cdot DF \cdot V_{rxn}\}}{n(monomer)\ \frac{DDA_{SM}}{100}} \quad \text{(S10)}$$

The term $n($monomer) can be developed using Equation (S11):

$$n(monomer) = \frac{m(sample)}{M_{av\_mono}} \qquad \text{(S11)}$$

in which m(sample) is the mass of chitosan starting material submitted to the reticulation reaction (g) and M_{av_mono} is the average molar mass of the monomer of that same starting material (g/mol).[1]

Finally, by merging Equation (S10) and (S11), we obtain Equation (S12) that can be directly used to determine LR_{HPLC} from our HPLC traces:

$$LR_{HPLC} = \left(\frac{100}{2}\right) \frac{n\,(GLA_{added}) - \{[GLA_{res\_HPLC}] \cdot DF \cdot V_{reaction}\}}{\left[\frac{m(sample)}{M_{av\_mono}}\right] \frac{DDA_{SM}}{100}} \qquad \text{(S12)}$$

b. Detailed example

As an example, we will calculate LR_{HPLC} for ChitoClear® HQG 800 / GLA/NH_2 1:4 (see Table S9, Entry 1 for complete experimental data).

When using the values from Table S9 for Equation (S12), we obtain

$$LR_{HPLC} = (\frac{100}{2}) \frac{0.0031\,mol - \{4.894 \cdot 10^{-5} mol \cdot L^{-1} \cdot 2.5 \cdot 0.04\,L\}}{\left[\frac{2.039g}{M_{av\_mono}}\right] \frac{DDA_{SM}}{100}}$$

[1] The average molar mass of monomer (*M_{av_mono}*) has been extensively described in Section 1d of this document,. This section also presents the the average molar masses of the monomer units for all three chitosan starting materials.

It has been established that, for ChitoClear® HQG 800:

DDA_{SM} = 69.4 ± 0.1   (see Table S1)
M_{av_mono} = 174,02 ± 0.04 g/mol (see Table S2)

Consequently,

$$LR_{HPLC} = \left(\frac{100}{2}\right) \frac{0.0031\ mol - \{4.894 \cdot 10^{-5} mol \cdot L^{-1} \cdot 2.5 \cdot 0.04\ L\}}{\left[\frac{2.039 g}{174.02 g \cdot mol^{-1}}\right] \frac{69.4}{100}} = 19.031$$

Uncertainties in the LR_{HPLC} values were calculated considering the uncertainties on DDA_{SM} and M_{av_mono} using the upper-lower bound method of uncertainty propagation.

For our example above, this gives us:

$$LR_{HPLC\_Min} = \left(\frac{100}{2}\right) \frac{0.0031\ mol - \{4.894 \cdot 10^{-5} mol \cdot L^{-1} \cdot 2.5 \cdot 0.04\ L\}}{\left[\frac{2.039 g}{(174.02 - 0.04)\ g \cdot mol^{-1}}\right] \frac{(69.4+0.1)}{100}} = 18.999$$

$$LR_{HPLC\_Max} = \left(\frac{100}{2}\right) \frac{0.0031\ mol - \{4.894 \cdot 10^{-5} mol \cdot L^{-1} \cdot 2.5 \cdot 0.04\ L\}}{\left[\frac{2.039 g}{(174.02 + 0.04)\ g \cdot mol^{-1}}\right] \frac{(69.4-0.1)}{100}} = 19.063$$

Thus for this sample, the definitive value for LR_{HPLC} is

$$LR_{HPLC} = 19.03\ \pm 0.03$$

To calculate the LR_{HPLC} for the whole triplicate, we calculated the mean. Uncertainties were obtained by considering the maximum and minimum values for each term and calculating the standard deviation.

For example, for ChitoClear® HQG 800 / GLA/NH_2 1:4 (Table S9, Entry 1,2 and 3)

LR_{HPLC} = 19.03 ± 0.03 (Table S9, Entry 1)
LR_{HPLC} = 18.55 ± 0.03 (Table S9, Entry 2)
LR_{HPLC} = 19.22 ± 0.03 (Table S9, Entry 3)

$$\mu = \frac{19.03 + 18.55 + 19.22}{3} = 18.93$$

$$\sigma = \sqrt{\frac{(18.93 - 19.03)^2 + (18.93 - 19.00)^2 + (18.93 - 19.06)^2 + (18.93 - 18.55)^2 + (18.93 - 18.52)^2 + (18.93 - 18.58)^2 + (18.93 - 19.22)^2 + (18.93 - 19.19)^2 + (18.93 - 19.25)^2}{9}} = 0.3$$

Thus, for ChitoClear® HQG 800 / GLA/NH_2 1:4 the final value of LR_{HPLC} reported in Table 3 of the manuscript is :

LR_{HPLC} = 18.9 ± 0.3 (see Table S9)

c. Comple data

Table S7 HPLC analysis and determination of LR_{HPLC} for reticulated derivatives of ChitoClear® HQG 10

Entry	GLA : NH_2 ratio	Peak retention time (min)	Peak area	Area Ratio*	Sum of areas	*m*(sample) (g)	[GLA_{res_HPLC}] (mol/L)	DF	*n*(GLA_{added}) (mol)	LR_{HPLC} (%)	LR_{HPLC} (%) Mean of triplicate
1		4.040	185.7	2.7	688.1	2.0527	$4.313\ 10^{-5}$	2.5	0.0031	17.26 ± 0.05	
		4.497	502.4								
2	1 : 4	4.041	175.9	2.9	689.1	2.0614	$4.319\ 10^{-5}$	2.5	0.0031	17.19 ± 0.05	17.2 ± 0.1
		4.500	513.2								
3		4.047	182.7	2.9	709.0	2.0813	$4.437\ 10^{-5}$	2.5	0.0031	17.02 ± 0.05	
		4.508	526.3								
4		4.041	147.4	3.1	605.7	2.0675	$3.825\ 10^{-5}$	5	0.0062	34.3 ± 0.1	
		4.500	458.3								
5	1 : 2	4.038	144.0	3.1	591.3	2.0495	$3.740\ 10^{-5}$	5	0.0062	34.6 ± 0.1	34.6 ± 0.3
		4.497	447.3								
6		4.037	146.4	3.1	599.0	2.0313	$3.786\ 10^{-5}$	5	0.0062	34.9 ± 0.1	
		4.497	452.6								
7		4.029	190.3	2.9	750.0	2.0544	$4.679\ 10^{-5}$	25	0.0124	68.8 ± 0.2	
		4.490	559.7								
8	1 : 1	4.030	173.2	2.9	675.0	2.0854	$4.236\ 10^{-5}$	25	0.0124	67.8 ± 0.2	69 ± 1
		4.490	501.8								
9		4.034	224.1	3.0	904.7	1.993	$5.595\ 10^{-5}$	25	0.0124	70.9 ± 0.2	
		4.494	680.6								
10		4.035	204.5	3.4	894.7	2.0866	$5.536\ 10^{-5}$	2500	0.0248	105.7 ± 0.3	
		4.496	690.2								
11	2 : 1	4.033	231.5	3.4	1019.1	2.0325	$6.272\ 10^{-5}$	2500	0.0248	104.3 ± 0.3	106 ± 2
		4.493	787.6								
12		4.036	221.5	3.4	976.1	1.9911	$6.017\ 10^{-5}$	2500	0.0248	108.0 ± 0.3	
		4.497	754.6								

13	4 : 1	4.039	511.9	3.4	2249.7	2.0605	1.355 10^{-4}	5000	0.0496	125.0 ± 0.4	119 ± 4
		4.500	1737.8								
14		4.035	533.4	3.4	2353.4	2.0712	1.417 10^{-4}	5000	0.0496	117.5 ± 0.4	
		4.494	1820.2								
15		4.035	547.8	3.4	2415.9	2.0349	1.454 10^{-4}	5000	0.0496	115.4 ± 0.4	
		4.496	1868.1								

For all experiments in Table S7 :

DDA_{SM} = 75.0 ± 0.2 (see Table S1)

M_{av_mono} = 171,67 ± 0.08 g/mol (see Table S2)

V_{rxn} = 0.04 L

Calibration curve equation: Sum of areas = 1.69 10^7 [GLA_{res_HPLC}] – 40.8 (Correlation coefficient R^2 =1)

* **Area ratio**

μ = 3.1

σ = 0.2

coefficient of variation (*CV*) = 0.0788

Table S8 HPLC analysis and determination of LRHPLC for reticulated derivatives of ChitoClear® HQG 400

Entry	GLA : NH_2 ratio	Peak retention time (min)	Peak area	Area Ratio	Sum of areas	*m*(sample) (g)	[GLA_{res_HPLC}] (mol/L)	DF	*n*(GLA_{added}) (mol)	LR_{HPLC} (%)	LR_{HPLC} (%) Mean of triplicate
1	1 : 4	3.977	203.0	2.9	791.4	2.0442	4.839 10^{-5}	2.5	0.0031	16.7 ± 0.2	16.8 ± 0.3
		4.422	588.4								
2		3.983	209.2	2.9	817.3	2.058	4.989 10^{-5}	2.5	0.0031	16.6 ± 0.2	
		4.429	608.1								
3		3.986	218.9	2.9	852.9	1.9876	5.195 10^{-5}	2.5	0.0031	17.1 ± 0.2	
		4.432	634.0								
4	1 : 2	3.980	213.5	3.1	953.7	2.0009	5.778 10^{-5}	5	0.0062	34.0 ± 0.3	34.0 ± 0.3
		4.426	722.2								

5		3.984<br>4.431	252.8<br>787.0	3.1	1039.8	1.994	6.275 10^{-5}	5	0.0062	34.2 ± 0.3	
6		3.984<br>4.432	245.2<br>777.7	3.2	1022.9	2.0188	6.177 10^{-5}	5	0.0062	33.7 ± 0.3	
7	1 : 1	3.980<br>4.429	588.9<br>1947.6	3.3	2536.5	2.0252	1.493 10^{-4}	25	0.0124	66.6 ± 0.6	66 ± 1
8		3.981<br>4.429	647.6<br>2069.3	3.2	2716.9	2.0074	1.597 10^{-4}	25	0.0124	67.1 ± 0.6	
9		3.978<br>4.426	533.6<br>1737.5	3.3	2271.1	2.0636	1.339 10^{-4}	25	0.0124	65.4 ± 0.6	
10	2 : 1	3.980<br>4.430	280.6<br>934.8	3.3	1215.4	2.0663	7.290 10^{-5}	2500	0.0248	93.3 ± 0.9	94 ± 1
11		3.985<br>4.434	274.2<br>920.0	3.4	1194.2	2.0532	7.168 10^{-5}	2500	0.0248	94.5 ± 0.9	
12		3.986<br>4.435	295.1<br>992.2	3.4	1287.3	1.9843	7.706 10^{-5}	2500	0.0248	94.8 ± 0.9	
13	4 : 1	3.987<br>4.437	578,8<br>1834.2	3.2	2413,0	2.0146	1.421 10^{-4}	5000	0.0496	116 ± 1	116 ± 3
14		3.992<br>4.443	557.9<br>1759.2	3.2	2317,1	2.0548	1.370 10^{-4}	5000	0.0496	119 ± 1	
15		3.996<br>4.447	581.2<br>1872.7	3.2	2453,9	2.0260	1.445$10^{-4}$	5000	0.0496	112 ± 1	

For all experiments in Table S8 :

DDA_{SM} = 77.5 ± 0.6 (see Table S1)

M_{av_mono} = 170,6 ± 0.3 g/mol (see Table S2)

V_{rxn} = 0.04 L

Calibration curve equation: Sum of areas = 1.73 10^7 [GLA_{res_HPLC}] – 45.8 (Correlation coefficient R^2 =1)

* **Area ratio**

μ = 3.2

σ = 0.1

coefficient of variation (CV) = 0.0545

Table S9 HPLC analysis and determination of LRHPLC for reticulated derivatives of ChitoClear® HQG 800

Entry	GLA/NH_2 ratio	Peak reten-tion time (min)	Peak area	Area Ra-tio	Sum of areas	*m*(sample) (g)	$[GLA_{res\_HPLC}]$ (mol/L)	DF	$n(GLA_{added})$ (mol)	LR_{HPLC} (%)	LR_{HPLC} (%) Mean of triplicate
1	1 : 4	3.998	207.7	2.8	788.6	2.039	$4.894\ 10^{-5}$	2.5	0.0031	19.03 ± 0.03	18.9 ± 0.3
		4.447	580.9								
2		3.997	189.3	2.8	717.2	2.0918	$4.474\ 10^{-5}$	2.5	0.0031	18.55 ± 0.03	
		4.446	527.9								
3		3.999	202.1	2.8	761.2	2.0192	$4.733\ 10^{-5}$	2.5	0.0031	19.22 ± 0.03	
		4.449	559.1								
4	1 : 2	3.994	170.4	3.0	685.9	2.0188	$4.290\ 10^{-4}$	5	0.0062	38.45 ± 0.06	38.4 ± 0.2
		4.444	515.5								
5		3.992	168.5	3.0	670.5	2.0323	$4.199\ 10^{-5}$	5	0.0062	38.19 ± 0.06	
		4.443	502.0								
6		4.001	165.1	3.0	665.3	2.0055	$4.169\ 10^{-5}$	5	0.0062	38.71 ± 0.06	
		4.453	500.2								
7	1 : 1	3.995	210.0	3.0	804.4	2.0897	$4.987\ 10^{-5}$	25	0.0124	74.1 ± 0.1	76 ± 1
		4.451	594.4								
8		3.994	271.1	3.0	1071.5	2.0029	$6.558\ 10^{-5}$	25	0.0124	77.2 ± 0.1	
		4.446	800.4								
9		3.996	246.6	3.1	999.4	2.0203	$6.134\ 10^{-5}$	25	0.0124	76.6 ± 0.1	
		4.448	752.8								
10	2 : 1	3.996	216.4	3.3	933.5	2.0089	$5.746\ 10^{-5}$	2500	0.0248	118.9 ± 0.2	118.4 ± 0.4
		4.448	717.1								
11		3.999	229.9	3.3	993.3	1.982	$6.098\ 10^{-5}$	2500	0.0248	118.3 ± 0.2	
		4.451	763.4								
12		4.002	201.8	3.3	873.7	2.0602	$5.395\ 10^{-5}$	2500	0.0248	118.09 ± 0.2	
		4.456	671.9								

13	4 : 1	4.000	561.9	3.6	2447.6	2.0806	1.465 10^{-4}	5000	0.0496	122.3 ± 0.2	121 ± 2
		4.452	1885.7								
14		4.002	568.6	3.4	2479.0	2.0421	1.484 10^{-4}	5000	0.0496	122.3 ± 0.2	
		4.455	1910.4								
15		4.003	592.8	3.4	2585.2	1.9882	1.546 10^{-4}	5000	0.0496	117.8 ± 0.2	
		4.455	1992.4								

For all experiments in Table S9 :

DDA_{SM} = 69.4 ± 0.1 (see Table S1)
M_{av_mono} = 174,02 ± 0.04 g/mol (see Table S2)
V_{rxn} = 0.04 L
Calibration curve equation: Sum of areas = 1.70 10^7 $[GLA_{res\_HPLC}]$ – 43.4 (Correlation coefficient R^2 =1)

* **Area ratio**
μ = 3.1
σ = 0.2
coefficient of variation (CV) = 0.0796

d. Example of chromatogram

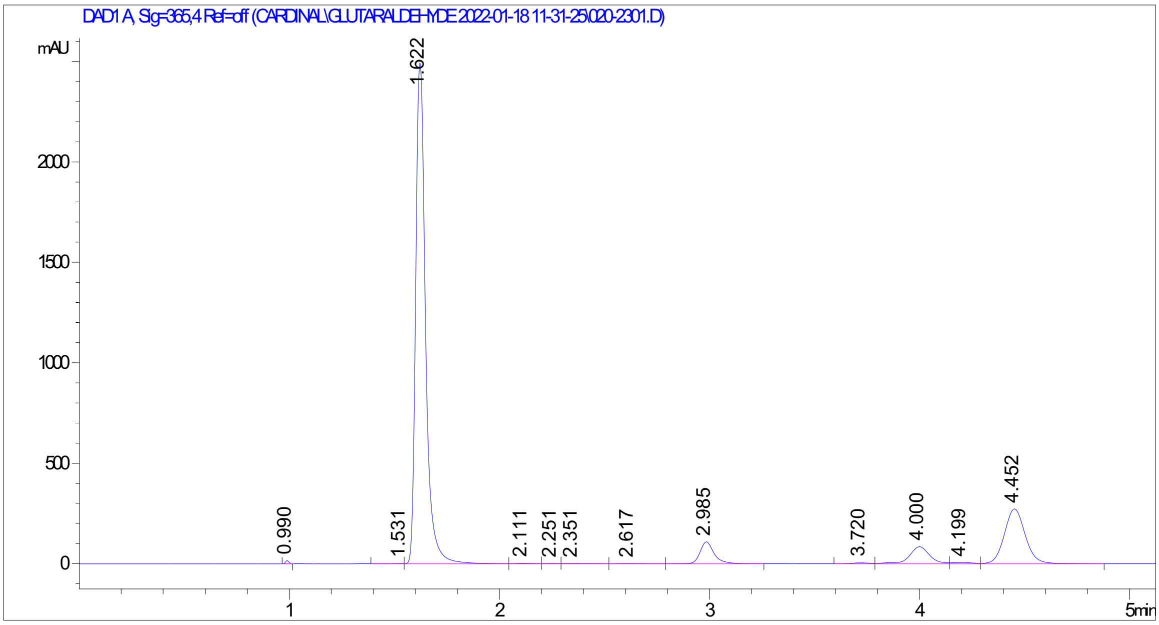

Figure S5. Chromatogram of the reaction medium (treated with DNPH) for the reticulation of ChitoClear® HQG 800 / GLA/NH_2 1:4 (Table S9, Entry 13). Peaks at 4.000 min and 4.452 min correspond to GLA(DNPH)$_2$. Peak at 1.622 min is attributed to unreacted excess DNPH. Peak at 2.985 min corresponds to an impurity that was found in the DNPH reagent. Total run time = 5 min.

5. FTIR spectra of chitosan resins

FTIR spectra of the three series of chitosan resins (the starting materials and all the reticulated derivatives) are shown in Figures S6–S8. In all cases, three bands were used to confirm the formation of the imide functionalities between glutaraldehyde and chitosan, accordingly to previous studies (refs. 31–32 of the main manuscript). The first band around 2940 cm^{-1} is attributed to the CH stretching vibration in the methylene groups. The second band around 1560 cm^{-1} is associated with ethylenic vibration. In both cases, those two bands become more defined and intense as the level of reticulation (along with the number of glutaraldehyde unit incorporated in the structure) increases. The third band around 1655 cm^{-1} is attributed to the C=N bond of the imine functionality. It is a bit more challenging to observe because unmodified chitosan shows a strong peak around 1645 cm^{-1} associated with the residual N-acetyl groups (C=O stretching of amide I). However, for our three series of reticulated chitosan, we observed that the maxima of the peak in this region of the spectra was drifting towards the higher frequencies as the level of reticulation increases, suggesting that the imine functionality makes a larger contribution.

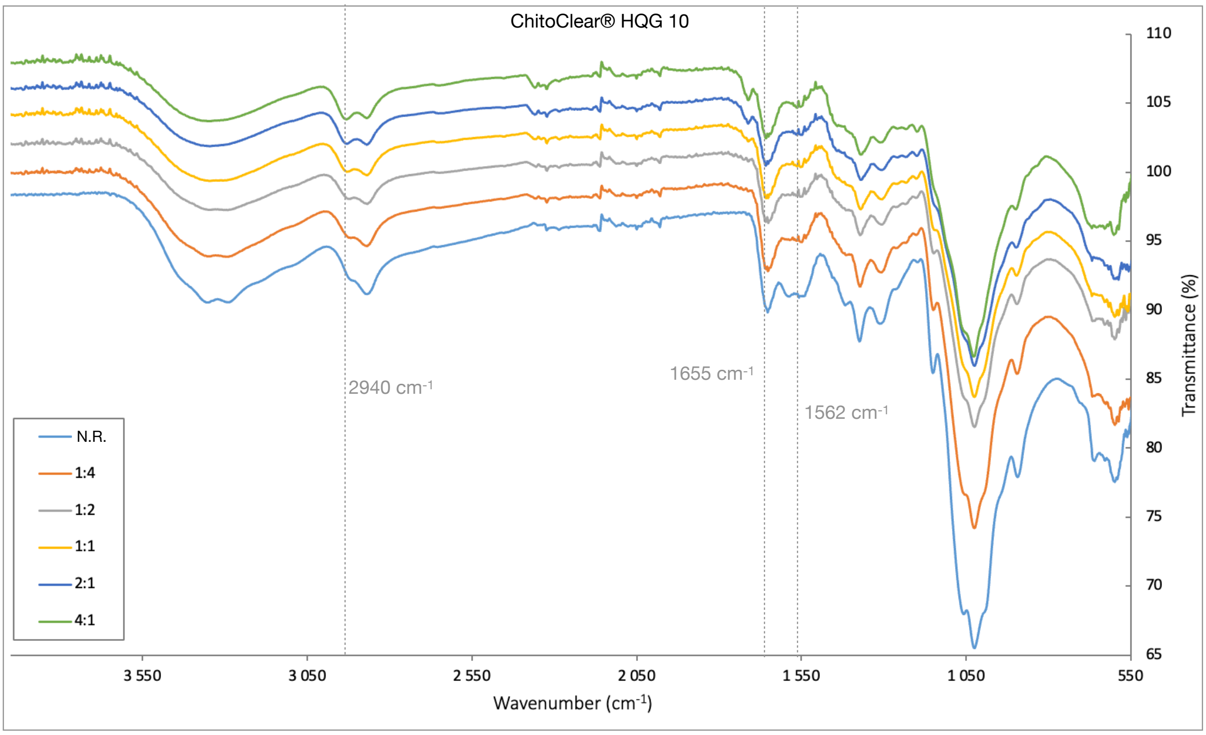

Figure S6. FTIR spectra of ChitoClear® HQG 10 before reticulation (N.R. = non reticulated; pale blue trace; bottom trace) and after reticulation with various GLA/NH_2 ratios (1:4, 1:2, 1:1, 2:1 and 4:1; 2nd to 6th traces from bottom).

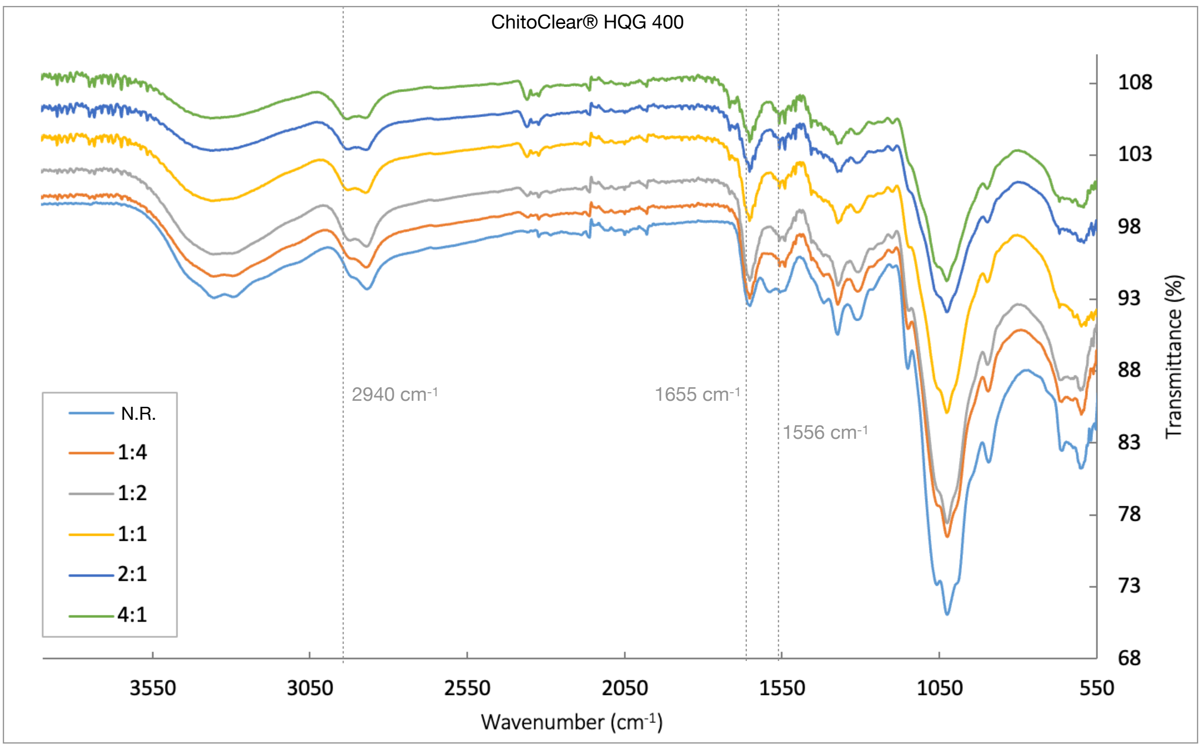

Figure S7. FTIR spectra of ChitoClear® HQG 400 before reticulation (N.R. = non reticulated; pale blue trace; bottom trace) and after reticulation with various GLA/NH_2 ratios (1:4, 1:2, 1:1, 2:1 and 4:1; 2nd to 6th traces from bottom).

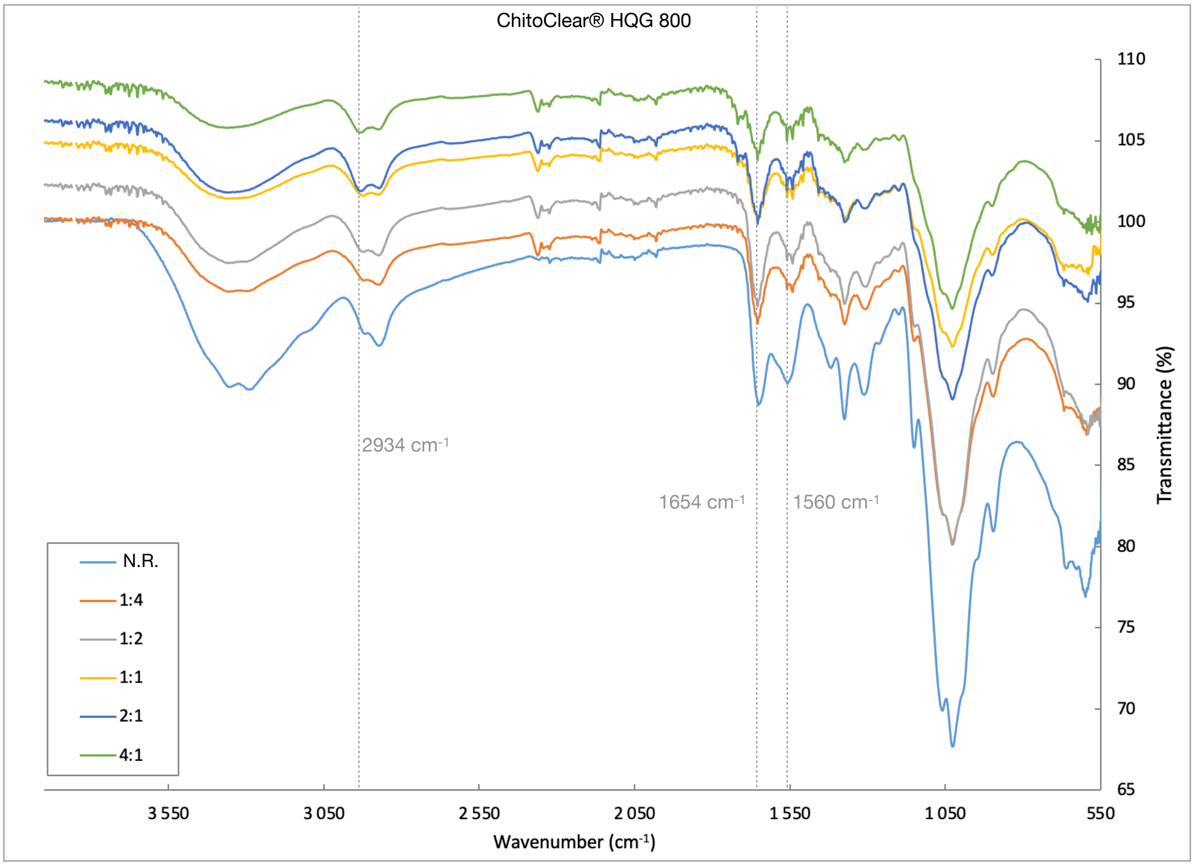

Figure S8. FTIR spectra of ChitoClear® HQG 800 before reticulation (N.R. = non reticulated; pale blue trace; bottom trace) and after reticulation with various GLA/NH_2 ratios (1:4, 1:2, 1:1, 2:1 and 4:1; 2nd to 6th traces from bottom).
